# Carvedilol for Prevention of Atrial Fibrillation after Cardiac Surgery: A Meta-Analysis

**DOI:** 10.1371/journal.pone.0094005

**Published:** 2014-04-04

**Authors:** Hui-Shan Wang, Zeng-Wei Wang, Zong-Tao Yin

**Affiliations:** Department of Cardiovascular Surgery, Shenyang Northern Hospital, Shenyang, Liaoning Province, China; Medical University Innsbruck, Austria

## Abstract

**Background:**

Postoperative atrial fibrillation (POAF) remains the most common complication after cardiac surgery. Current guidelines recommend β-blockers to prevent POAF. Carvedilol is a non-selective β-adrenergic blocker with anti-inflammatory, antioxidant, and multiple cationic channel blocking properties. These unique properties of carvedilol have generated interest in its use as a prophylaxis for POAF.

**Objective:**

To investigate the efficacy of carvedilol in preventing POAF.

**Methods:**

PubMed from the inception to September 2013 was searched for studies assessing the effect of carvedilol on POAF occurrence. Pooled relative risk (RR) with 95% confidence interval (CI) was calculated using random- or fixed-effect models when appropriate. Six comparative trials (three randomized controlled trials and three nonrandomized controlled trials) including 765 participants met the inclusion criteria.

**Results:**

Carvedilol was associated with a significant reduction in POAF (relative risk [RR] 0.49, 95% confidence interval [CI] 0.37 to 0.64, p<0.001). Subgroup analyses yielded similar results. In a subgroup analysis, carvedilol appeared to be superior to metoprolol for the prevention of POAF (RR 0.51, 95% CI 0.37 to 0.70, p<0.001). No evidence of heterogeneity was observed.

**Conclusions:**

In conclusion, carvedilol may effectively reduce the incidence of POAF in patients undergoing cardiac surgery. It appeared to be superior to metoprolol. A large-scale, well-designed randomized controlled trial is needed to conclusively answer the question regarding the utility of carvedilol in the prevention of POAF.

## Introduction

Despite significant advances in anesthetic and surgical techniques, postoperative atrial fibrillation (POAF) remains the most common complication after cardiac surgery [1]–[3]. The incidence of POAF varies from 11% to 40%, depending on the definition and the method of monitoring [1]–[3]. Although this arrhythmia is usually benign and self-limiting, it may result in hemodynamic instability, a longer hospital stay, and increased health care costs [1]–[3]. Given the clinical consequences attributable to POAF, its prevention is of great importance. To date, many pharmacologic approaches have been attempted to prevent POAF, for example, β-blockers, amiodarone, and magnesium [4]. Most reviews reflect a growing consensus in favor of the prophylactic administration of β-blockers for cardiac surgery patients [5]. In addition, updated American College of Cardiology/American Heart Association (ACC/AHA) 2006 guidelines recommend β-blockers for the prevention of POAF [6].

Despite the extensive studies, the exact pathophysiology of POAF is for the moment far from being fully elucidated [1]–[3]. A growing body of evidence suggests that markers of inflammation and oxidative injury are elevated in atrial fibrillation patients [7]–[10]. Carvedilol, a non-selective β-adrenergic blocking agent approved for use in heart failure cases, has a number of ancillary activities including anti-inflammatory and antioxidant properties [11], [12]. Moreover, unlike other beta-blockers, carvedilol antagonizes the rapid-depolarizing sodium channel, the human ether-a-go-go-related gene potassium channel, and the L-type calcium channel [11], [12], which suggests a pharmacologic profile similar to amiodarone, a proven anti-arrhythmic agent for the prevention of POAF [13]. Theoretically, this should reduce the incidence of arrhythmia, including POAF. All these properties of carvedilol have generated interest in its use as a prophylactic agent for POAF. Recently, several relevant studies regarding prophylactic carvedilol in preventing POAF have been published [14]–[19]. However, the role of carvedilol in preventing POAF remains unknown. We therefore undertook a meta-analysis of published studies to the efficacy of carvedilol in preventing POAF for adult patients undergoing cardiac surgery.

## Methods

### Literature search and inclusion criteria

Two investigators searched PubMed database for relevant articles published up to September 2013. The initial search terms were carvedilol and atrial fibrillation. No language restriction was imposed. In addition, the reference lists of identified studies were manually checked to include other potentially eligible trials. This process was performed iteratively until no additional articles could be identified.

The following inclusive selection criteria were applied: (i) study design: comparative trial; (ii) study population: adult patients undergoing cardiac surgery; (iii) intervention: carvedilol (no matter what regimen applied); (iv) comparison intervention: control (placebo or other beta-blockers) and (v) outcome measure: the incidence of POAF.

### Data extraction and outcome measures

Two investigators independently extracted the following data from each trial: first author, publication year, number of patients (carvedilol/control), patient characteristic, regimen of intervention (carvedilol/control), definition and monitoring of POAF, study design, the incidence of POAF, and length of hospital stay (LOS). Extracted data were entered into a standardized Excel file. The primary outcome was the incidence of POAF. Secondary outcome included LOS.

### Statistical analysis

Differences were expressed as relative risks (RRs) with 95% confidence intervals (CIs) for dichotomous outcomes, and weighted mean differences (WMDs) with 95% CIs for continuous outcomes. Heterogeneity across studies was tested by using the I^2^ statistic, which was a quantitative measure of inconsistency across studies. Studies with an I^2^ statistic of 25% to 50% were considered to have low heterogeneity, those with an I^2^ statistic of 50% to 75% were considered to have moderate heterogeneity, and those with an I^2^ statistic of >75% were considered to have a high degree of heterogeneity [20]. An I^2^ value greater than 50% indicates significant heterogeneity [21]. A fixed-effects model was used (I^2^≤50%), and a random-effects model was used in the case of significant heterogeneity (I^2^>50%). We further conducted subgroup analyses according to type of control, surgery type, and study design. We also investigated the influence of a single study on the overall risk estimate by omitting one study in each turn. We did not assess publication bias [22], because the pooled estimate included fewer than ten trials. A p value <0.05 was considered statistically significant. All statistical analyses were performed using Stata version 11.0 (Stata Corporation, College Station, Texas, USA).

## Results

### Study identification and selection

**Figure 1:**
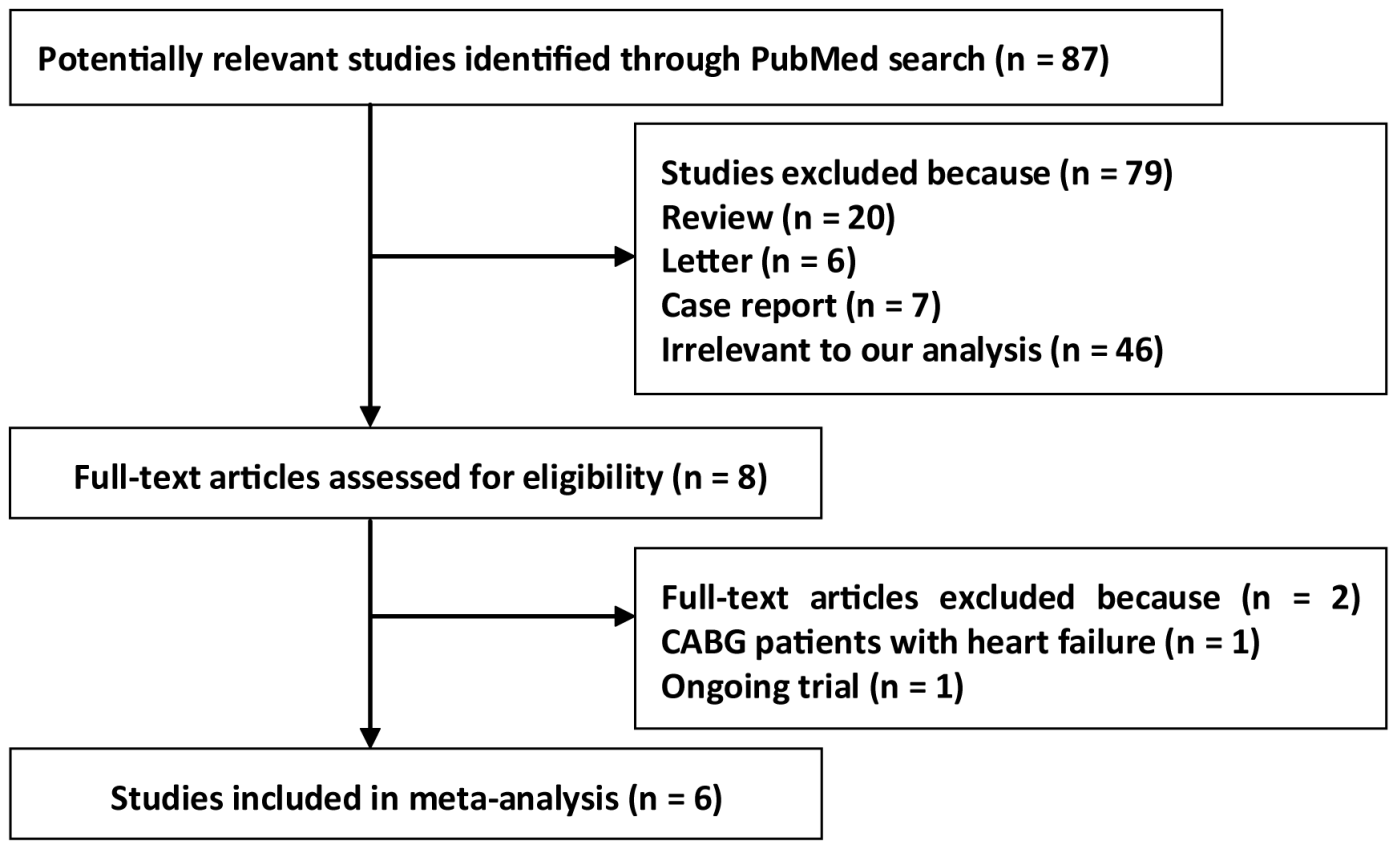
Selection process for clinical trials.

The initial search yielded 87 relevant publications of which 79 were excluded for various reasons (review, letter, case report, or irrelevant to the current analysis) based on the titles and abstracts. The remaining eight were then retrieved for full text review, two of them were also excluded because one was focused in patients undergoing coronary bypass graft with heart failure and one was currently ongoing [23], [24]. Thus, six studies were included in the final analysis [14]–[19]. The flowchart of studies included in meta-analysis was shown in Figure 1.

### Study characteristics

The basic characteristics of studies included in the meta-analysis are shown in Table 1. These studies were published between 2003 and 2010. The sample size of these studies ranged from 53 to 207 (total 765). Four studies in this meta-analysis enrolled patients undergoing coronary artery bypass grafting (CABG) only [15]–[18]. The remaining two included patients undergoing CABG and/or valve surgery [14], [19]. Carvedilol was administered orally by different regimens and formulations. Timing of initiation for carvedilol prophylaxis was 3–10 days before the surgery in the preoperative prophylaxis studies [15], [16], [19] and within 24 hours of surgery in the postoperative group [17], [18]. Definition of POAF in terms of duration varied among the studies. All the patients were monitored using electrocardiography.

**Table 1:** Characteristics of studies included in the meta-analysis.

Study (Reference)	Sample size (Carvedilol/Control)	Patient characteristic	Mean age (year)/Male (%)	Regimen of intervention		POAF		LOS (days)		Study design
				Carvedilol	Control	Carvedilol	Control	Carvedilol	Control	
Merritt 2003 [14]	115(26/89)	Adult patients undergoing CABG and/or VS	60.3/NA	NA	Metoprolol/atenolol	2/26	28/89	5.9±1.9	6.9±4.5	Non-RCT
Haghjoo 2007 [15]	120(60/60)	Adult patients undergoing CABG	61/52.5	6.25 mg twice daily, oral, starting from 10 days before surgery, then increasing until to the maximum	Metoprolol 25 mg twice daily, oral, starting from 10 days before surgery, then increasing until to the maximum	9/60	20/60	NA	NA	RCT
Acikel 2008 [16]	110(55/55)	Adult patients undergoing CABG	60/71.8	12.5 mg twice daily, starting on 3 days prior to surgery, lasting to the morning of surgery, then titrating according to hemodynamic responses after CABG	Metoprolol 50 mg twice daily, starting on 3 days prior to surgery, lasting to the morning of surgery, then titrating according to hemodynamic responses after CABG	9/55	20/55	NA	NA	RCT
Tsuboi 2008 [17]	160(80/80)	Adult patients undergoing CABG	66.5/70.6	5 or 10 mg/day, oral, starting on postoperative days 1 or 2, then increasing until to the maximum	Placebo	12/80	27/80	17.0±6.2	22.0±12.3	Non-RCT
Yoshioka 2009 [18]	53(31/22)	Adult patients undergoing CABG	67/68	2.5 mg/day, oral, starting on postoperative days 1 or 2	Placebo	4/31	7/22	NA	NA	Non-RCT
Ozaydin 2013 [19]	207(104/103)	Adult patients undergoing CABG and/or VS	63/72.5	6.25 mg twice daily, starting from 7 days before surgery, if not tolerated, a 3.125 mg twice daily dose was given	Metoprolol 50 mg once daily dose, starting from 7 days before surgery, if not tolerated, a 25 mg twice daily dose was given	25/104	37/103	NA	NA	RCT

CABG, coronary artery bypass grafting; LOS, length of hospital stay; NA, no data available; POAF, postoperative atrial fibrillation; RCT, randomized controlled trial; VS, valve surgery.

### Primary outcome: POAF

**Figure 2:**
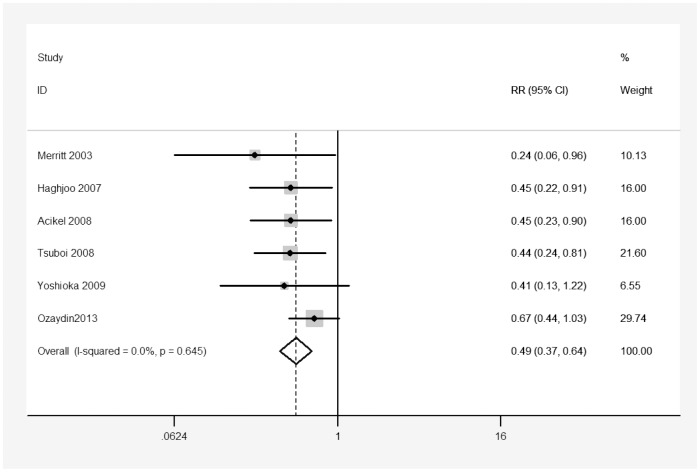
Effect of carvedilol versus control on the incidence of postoperative atrial fibrillation.

The definition and monitoring of POAF in each trial are summarized in Table 2. Overall, six studies including 765 patients were included in this analysis (356 in the carvedilol group and 409 in the control group). Meta-analysis of six studies using a fixed-effects model suggested that carvedilol significantly reduced the incidence of POAF in patients undergoing cardiac surgery compared with control (RR 0.49, 95% CI 0.37 to 0.64, p<0.001; Figure 2). There was no heterogeneity among the studies (I^2^ = 0%, heterogeneity p = 0.645; Figure 2).

**Table 2:** Definition and monitoring of POAF.

Study (Reference)	Definition of POAF	Monitoring of POAF
Merritt 2003 [14]	NA	NA
Haghjoo 2007 [15]	Absent P wave before the QRS complex together with irregular ventricular rhythm on the rhythm strips, lasting longer than 5 minute.	ECG and 12-lead ECG were need to confirm
Acikel 2008 [16]	An irregular rhythm with no prominent P waves lasting 30 s or more	Automated arrhythmia detectors in cardiac ICU, and simultaneous telemetric display of ECG in the ward
Tsuboi 2008 [17]	Absent consistent P waves before each QRS complex and an irregular ventricular rate and as episodes of atrial fibrillation that persisted for over 10 min.	12-lead ECG
Yoshioka 2009 [18]	Lasted more than 5 minutes or required intervention for angina or hemodynamic compromise, or any episode that required intervention for angina or hemodynamic compromise.	Monitoring system on a rhythm strip or 12-lead ECG
Ozaydin 2013 [19]	An irregular rhythm with the absence of discrete P-waves lasting 5 min during hospitalization	Continuous ECG monitoring and all-day Holter

ECG, electrocardiogram; NA, no data available; POAF, postoperative atrial fibrillation; ICU, intensive care unit.

**Figure 3:**
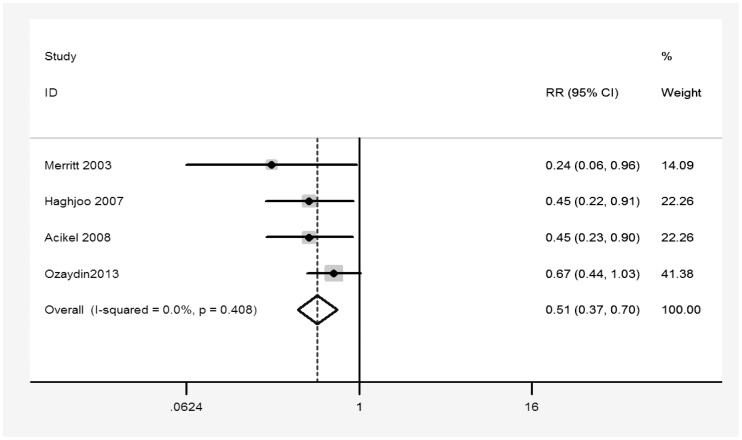
Effect of carvedilol versus metoprolol on the incidence of postoperative atrial fibrillation.

Then we further conducted subgroup analyses based on type of control (metoprolol vs. placebo), surgery type (CABG and/or valve surgery vs. CABG only), and study design (randomized trials vs. nonrandomized trials). Table 3 shows the results of subgroup analyses for POAF. The results suggested that carvedilol appeared to be superior to metoprolol for the prevention of POAF (RR 0.51, 95% CI 0.37 to 0.70, p<0.001; Figure 3). No evidence of heterogeneity was observed in subgroup analysis. Influence analysis suggested exclusion of any single study did not materially alter the overall combined RR, with a range from 0.41 (0.29 to 0.59) to 0.52 (0.39 to 0.68), which adds robustness to our results.

**Table 3:** Results of subgroup analyses for POAF.

Subgroup analysis	n (N)	Carvedilol	Control	OR (95% CI)	p value	I^2^(%)	Heterogeneity p
Study design							
RCTs [15], [16], [19]	3 (437)	43/219	77/218	0.56 (0.40–0.77)	<0.001	0	0.489
Non-RCTs [14], [17], [18]	3 (328)	18/137	62/191	0.38 (0.23–0.64)	<0.001	0	0.723
Surgery type							
CABG and/or valve surgery [14], [19]	2 (322)	27/130	65/192	0.56 (0.37–0.85)	0.007	51.7	0.15
CABG only [15]–[18]	4 (443)	34/226	74/217	0.44 (0.31–0.64)	<0.001	0	0.999
Type of comparison							
Metoprolol [14]–[16], [19]	4 (552)	45/245	105/307	0.51 (0.37–0.70)	<0.001	0	0.408
Placebo [17], [18]	2 (213)	16/111	34/102	0.44 (0.26–0.74)	0.002	0	0.886

CABG, coronary artery bypass grafting; RCT, randomized controlled trial; n, number of patients; N, number of trials.

### Secondary outcome: LOS

Two trials reported the effect of carvedilol on LOS and provided available data (expressed as mean ± standard deviation) with a total of 275 patients. The combined analysis using a random-effects model showed that carvedilol did not significantly reduce LOS (WMD −2.75, 95% CI −6.64 to 1.14, p = 0.17), with a high degree of heterogeneity between the trials (I^2^ = 82.9%, heterogeneity p = 0.016).

### Publication bias

Publication bias was not assessed because of the limited number (below 10) of studies included in the analysis.

## Discussion

Meta-analysis of all six included studies using a fixed-effects model illustrates that carvedilol may effectively reduce the incidence of POAF in adult patients undergoing cardiac surgery.

The mechanisms that carvedilol reduces the incidence of POAF are not entirely known. However, there is now an increasing body of evidences that oxidative stress [25], and inflammation [26], [27], and increased sympathetic activation [28] are involved in the pathogenesis of POAF. Carvedilol is a β blocker with antioxidant and anti-inflammatory properties [11], [12], and reduces sympathetic activity [29]. From a pathophysiological point of view, it is plausible that the abovementioned properties of carvedilol might result in the favorable effect on the prevention of POAF.

Recently, Khan et al carried out a meta-analysis of randomized controlled trials and confirmed the efficacy of prophylactic beta-blockers against POAF [30]. Both the Khan meta-analysis and our meta-analysis showed that carvedilol appeared to be more effective than metoprolol for the prevention of POAF. Compared with metoprolol, carvedilol has been shown to increase the levels of antioxidant enzymes (superoxide dismutase and glutathione peroxidase). Moreover, carvedilol may have direct antiarrhythmic profile through electrophysiological traits, since it blocks multiple cationic channels (Na^+^, K^+^, and Ca^2+^) [11], [12]. These properties of carvedilol, which are not equally shared by metoprolol, may partly explained superior efficacy of carvedilol in preventing POAF. In addition, numerous trials indicate that carvedilol is better than conventional β1-selective β blockers on reducing sympathetic activation, a risk factor for atrial fibrillation [28], [29].

In this meta-analysis carvedilol did not significantly reduce the LOS. The total incidence of POAF is 26.1% (200 of 765), less than one-third of patients develop POAF and still fewer develop prolonged atrial fibrillation, so the effect of carvedilol on LOS in patients prone to atrial fibrillation would have to be very large to be able to detect an effect of LOS in the total population. In addition, a relatively small number of samples (only two studies) provided available data on LOS, additional studies or data are warranted.

One problem with the use of carvedilol to prevent POAF is that the majority of patients does not develop POAF after cardiac surgery but would still be exposed to possible side effects. In this meta-analysis, two trials reported carvedilol was well tolerated and side effects attributable to carvedilol were detected. And one trial reported complication rates were similar between carvedilol and control groups, including postoperative myocardial infarction and renal dysfunction.

Several potential limitations of this meta-analysis merit consideration. First, our study included only six studies and some of them have a modest sample size. Overestimation of the treatment effect is more likely in smaller studies compared with larger samples. Second, our analysis is based on six clinical studies, and half of them were non-randomized controlled trials. The targeted population, adopted carvedilol protocols, type of control, and study design differed among the included studies. These factors may result in the heterogeneity and have potential impact on our results. Furthermore, these studies lack homogeneity in both the method of postoperative monitoring and in their definition of POAF. This may lead to potential underestimation and/or overestimation of the true incidence of POAF. Finally, it was possible that some missing and unpublished data may lead to bias in effect size.

In conclusion, despite its various limitations, our study is clinically valuable because it revealed that carvedilol leads to lower incidence of POAF than control and appears to be superior to metoprolol as the current study clearly delineated. Carvedilol may effectively reduce the incidence of POAF in patients undergoing cardiac surgery. On the basis of this encouraging finding, we believe that research on the field is promising and should be continued. At least the ongoing COMPACT [24], which is a prospective, multi-center, randomized, open-label, active-controlled trial, will answer the question of whether or not carvedilol is more superior to metoprolol in preventing POAF in patients undergoing CABG.

## Supporting Information

Checklist S1 PRISMA Checklist.
(DOC)
